# Multicentre, prospective, randomised, open-label, blinded end point trial of the efficacy of allopurinol therapy in improving cardiovascular outcomes in patients with ischaemic heart disease: protocol of the ALL-HEART study

**DOI:** 10.1136/bmjopen-2016-013774

**Published:** 2016-09-08

**Authors:** Isla S Mackenzie, Ian Ford, Andrew Walker, Chris Hawkey, Alan Begg, Anthony Avery, Jaspal Taggar, Li Wei, Allan D Struthers, Thomas M MacDonald

**Affiliations:** 1 Medicines Monitoring Unit (MEMO) and Hypertension Research Centre, Division of Molecular and Clinical Medicine, University of Dundee and Ninewells Hospital, Dundee, UK; 2 Glasgow Clinical Trials Unit, Robertson Centre for Biostatistics, University of Glasgow, Glasgow, UK; 3 Nottingham Centre for Digestive Disorders, Nottingham University Hospitals NHS Trust, Nottingham, UK; 4 Townhead Medical Practice, Montrose, UK; 5 School of Medicine, University of Nottingham, Nottingham, UK; 6 Division of Primary Care, School of Medicine, University of Nottingham, Nottingham, UK; 7 Robertson Centre for Biostatistics, Glasgow Clinical Trials Unit, University of Glasgow, Glasgow, UK; 8 Division of Molecular and Clinical Medicine, University of Dundee and Ninewells Hospital, Dundee, UK

**Keywords:** allopurinol, cardiovascular outcomes, quality of life, uric acid

## Abstract

**Introduction:**

Ischaemic heart disease (IHD) is one of the most common causes of death in the UK and treatment of patients with IHD costs the National Health System (NHS) billions of pounds each year. Allopurinol is a xanthine oxidase inhibitor used to prevent gout that also has several positive effects on the cardiovascular system. The ALL-HEART study aims to determine whether allopurinol improves cardiovascular outcomes in patients with IHD.

**Methods and analysis:**

The ALL-HEART study is a multicentre, controlled, prospective, randomised, open-label blinded end point (PROBE) trial of allopurinol (up to 600 mg daily) versus no treatment in a 1:1 ratio, added to usual care, in 5215 patients aged 60 years and over with IHD. Patients are followed up by electronic record linkage and annual questionnaires for an average of 4 years. The primary outcome is the composite of non-fatal myocardial infarction, non-fatal stroke or cardiovascular death. Secondary outcomes include all-cause mortality, quality of life and cost-effectiveness of allopurinol. The study will end when 631 adjudicated primary outcomes have occurred. The study is powered at 80% to detect a 20% reduction in the primary end point for the intervention. Patient recruitment to the ALL-HEART study started in February 2014.

**Ethics and dissemination:**

The study received ethical approval from the East of Scotland Research Ethics Service (EoSRES) REC 2 (13/ES/0104). The study is event-driven and results are expected after 2019. Results will be reported in peer-reviewed journals and at scientific meetings. Results will also be disseminated to guideline committees, NHS organisations and patient groups.

**Trial registration number:**

32017426, pre-results.

**Strengths and limitations of this study**

- The ALL-HEART study is a large prospective randomised study designed to answer the question of whether allopurinol improves cardiovascular outcomes in patients with ischaemic heart disease.
- The trial is conducted within the normal UK healthcare setting, providing good external validity. The end point committee is blinded to treatment allocation.
- One limitation of the study is that it does not include patients with ischaemic heart disease who are younger than 60 years old.

## Introduction

The ALL-HEART study is an academic clinical trial that aims to address whether allopurinol added to usual therapy improves cardiovascular (CV) outcomes in patients with ischaemic heart disease (IHD).

IHD is one of the most common causes of death in men and women in the UK and most other developed countries.^1^ Although death rates from IHD have fallen in the past 10 years, largely due to reductions in smoking and improvements in treatment and secondary prevention, morbidity from IHD is increasing. Patients with IHD often have reduced quality of life due to symptoms, limitation of activities and psychological impact of the disease. IHD is usually treated with a combination of medications, some of which improve symptoms and some of which improve survival. In addition, interventional procedures such as angioplasty, coronary artery stenting and coronary artery bypass grafting are used in selected patients.

Allopurinol is a xanthine oxidase inhibitor that lowers uric acid levels and is licensed for the prevention of gout, but is not currently indicated for treating asymptomatic hyperuricaemia. Allopurinol is usually given in doses of 100–900 mg daily. Xanthine oxidase promotes oxidative stress which inactivates the antiatherosclerotic substance nitric oxide. Xanthine oxidase levels are raised in several conditions including coronary artery disease.^2^ Allopurinol has several beneficial effects in CV disease. It improves endothelial function in patients with heart failure,^3^ ^4^ type 2 diabetes^5^ and smokers,^6^ reduces left ventricular mass in patients with IHD^7^ and reduces left ventricular hypertrophy in patients with type 2 diabetes mellitus^8^ and chronic kidney disease.^9^ It also lowers blood pressure and decreases arterial stiffness and carotid intima-media thickness progression.^7^ ^10–13^ Allopurinol reduces the troponin rise in patients undergoing percutaneous coronary intervention following ST elevation myocardial infarction (MI), which is strong evidence that downstream ischaemic cardiomyocytes stay alive better during an ischaemic insult in the presence of allopurinol therapy.^14^ In patients with angina and angiographically documented coronary artery disease, allopurinol 600 mg daily has anti-ischaemic activity in that it increases exercise time and reduces chest pain.^15^

In earlier observational studies in patients with hyperuricaemia^16^ and heart failure,^17^ ^18^ allopurinol use was associated with reduced mortality. In recent case–control studies, allopurinol use was associated with a reduced risk of MI^19^ and a reduced risk of non-fatal MI.^20^ In a recent cohort study in patients with hyperuricaemia, patients on allopurinol treatment had a lower risk of MI, stroke or CV death and a lower risk of all-cause mortality compared with non-users of allopurinol.^21^ A study conducted in the UK Clinical Practice Research Datalink in patients aged over 65 years with hypertension showed that allopurinol use was associated with a significantly lower risk of stroke and cardiac events than in non-exposed control patients. In this study, treatment with higher dose allopurinol (defined as 300 mg daily) was associated with a significantly lower risk of stroke and cardiac events than treatment with a lower dose of allopurinol.^22^ In patients with hypertension and impaired renal function, allopurinol use was associated with a lower risk of developing CV disease and all-cause mortality.^23^ A long-term follow-up study of a 2-year randomised trial of 113 patients with chronic kidney disease showed that long-term treatment with allopurinol may slow the rate of progression of kidney disease and reduce CV risk.^24^ ^25^

Various mechanisms have been suggested as to how allopurinol may improve CV outcomes. Xanthine oxidase is a major source of reactive oxygen species.^26^ Allopurinol profoundly reduces oxidative stress by reducing superoxide anions and other free radicals, which reduces cardiac hypertrophy, increases tissue oxygenation and reduces atherosclerotic plaque rupture which is involved in MI.^27–30^ Allopurinol may also reduce cardiac afterload by improving arterial compliance through reduced wave reflection and improved endothelial function. It is not clear whether it is uric acid lowering or other effects of allopurinol that are important, although in one study comparing allopurinol with the uricosuric agent probenecid, effects on endothelial function were only seen with xanthine oxidase inhibition and not by reducing uric acid with probenecid.^27^ Finally, by inhibiting xanthine oxidase activity, allopurinol increases levels of hypoxanthine, which might increase ATP levels and thus energy availability to tissues. Extra ATP and oxygen availability produced by allopurinol might prevent downstream ischaemic cardiomyocytes from infarcting and thereby leading to heart failure during an ischaemic insult such as acute coronary syndrome (ACS).^31^ ^32^

Some studies have suggested that higher doses of allopurinol (often 600 mg daily) are necessary to achieve some of the positive CV effects,^6–8^ ^15^ ^22^ ^27^ which is why a dose of 600 mg daily was selected for participants with normal renal function at baseline for the current study. It has previously been demonstrated that this can be given safely in patients with angina.^15^

Most of the papers in this field still conclude that there is a need for large randomised controlled trials to answer the question of whether allopurinol is beneficial in patients with CV disease. The ALL-HEART study is the key outcome study that will provide the answer to this question in patients with IHD, in addition to providing robust data on any improvements in quality of life and an analysis of the health economics of the use of allopurinol in the UK National Health Service (NHS) in patients with IHD.

## Methods and analysis

### Objectives

The primary objective of the study is to determine whether the addition of allopurinol (up to 600 mg daily) to usual therapy improves CV outcomes. The secondary objectives are to determine the cost-effectiveness of adding allopurinol to usual therapy, to determine whether allopurinol improves quality of life and to determine the safety and tolerability of giving allopurinol to patients with IHD (without a history of gout).

### Outcomes

The primary outcome is the composite CV end point of non-fatal MI, non-fatal stroke or CV death. The secondary outcomes are non-fatal MI; non-fatal stroke; CV death; all-cause mortality; all CV hospitalisations; hospitalisation for ACS (includes hospitalisation for MI and for troponin-negative cardiac chest pain); coronary revascularisation; hospitalisation for ACS or coronary revascularisation, hospitalisation for heart failure, quality of life (EQ-5D and Seattle Angina Questionnaire); cost-effectiveness of allopurinol. Data will also be collected on the safety and tolerability of allopurinol in patients with IHD, in particular, discontinuations of allopurinol due to adverse events including gout flares and serious adverse skin reactions.

### Ethics and trial registration

The study received approval from the UK Medicines and Healthcare products Regulatory Agency (MHRA), local research and development departments and the national Caldicott guardian in Scotland. Patient recruitment to the study started on 7 February 2014. The trial was prospectively registered in ISRCTN (ISRCTN32017426; 16 August 2013) and EudraCT (2013-003559-39; 19 September 2013). Protocol amendments will be approved by the ethics committee and regulatory authorities as per current guidelines and will be communicated to investigators and the primary trial registry by the study team.

### Study design

The study is a multicentre, controlled, prospective, randomised, open-label, blinded end point (PROBE) superiority trial of allopurinol (up to 600 mg daily) versus no treatment added to usual therapy in patients aged 60 years and over with IHD. The study is end point driven and will end when the required number of adjudicated primary end points have occurred.

#### Number of participants

**Figure 1:**
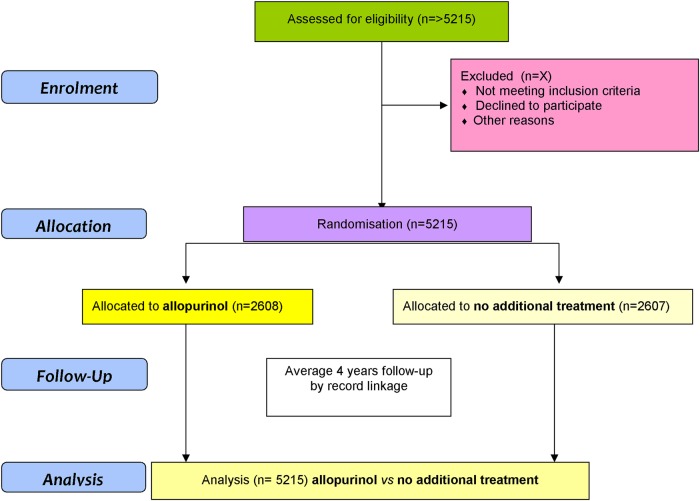
ALL-HEART study overview. In total, 5215 patients aged over 60 years with ischaemic heart disease will be randomised to receive allopurinol or no additional therapy, in addition to their usual care, then followed up for an expected average of 4 years for outcomes.

In total, 5215 patients will be randomised in a 1:1 ratio to allopurinol or no additional treatment (figure 1). Patients will be recruited from the UK. Approximately 290 primary care practices will be involved, and the study will be coordinated by two main study sites (Dundee, Scotland and Nottingham, England). Recruitment was initially expected to last for ∼2 years with an expected average follow-up period of 4 years. The recruitment phase was extended in 2016 and the follow-up phase may be extended depending on recruitment rates and event rates.

#### Identification of participants, screening visit and randomisation

Primary care practice lists are searched for suitable patients with IHD who are invited to participate. Other patients with IHD who volunteer to take part in the trial may be included. Patients may also be invited to participate from secondary care clinics and consented research databases. At a screening visit, written informed consent is taken by a research nurse (or doctor), inclusion and exclusion criteria checked (box 1) and blood samples taken for baseline full blood count, urea and electrolytes, creatinine, estimated glomerular filtration rate (eGFR) and urate. Baseline demographics, medical history, CV risk factors, medication, blood pressure and height and weight data are collected.

Box 1 **Inclusion and exclusion criteria**

*Inclusion criteria:*

1. Male or female patients aged 60 years and over.
2. Ischaemic heart disease (IHD) defined as a diagnosis of angina or myocardial infarction at any time or other evidence of IHD (investigator opinion).

*Exclusion criteria:*

1. History of gout.
*Known severe renal impairment (estimated glomerular filtration rate (eGFR) <30 mL/min).
3. Moderate-to-severe heart failure (New York Heart Association III–IV).
4. Significant hepatic disease (eg, alanine transaminase>3× upper limit of normal, cirrhosis, ascites; investigator opinion).
5. Patients currently taking part in another interventional clinical trial of an investigational medicinal product or medical device (or taken part in one within the past 3 months).
6. Previous allergy to allopurinol.
7. Previous serious adverse cutaneous (skin) reaction to any drug (eg, Stevens Johnson syndrome, toxic epidermal necrolysis, hospitalisation due to skin reaction to drug; investigator opinion).
8. Patients already taking urate-lowering therapy (including allopurinol, febuxostat, sulfinpyrazone, benzbromarone, probenecid, rasburicase).
9. Patients taking azathioprine, mercaptopurine, ciclosporin or theophylline.
10. Malignancy (except non-metastatic, non-melanoma skin cancers, cervical in situ carcinoma, breast ductal carcinoma in situ, or stage 1 prostate carcinoma) within the past 5 years (investigator opinion).

*Exclusion criterion 2 was previously ‘known renal impairment eGFR<60 mL/min’ for patients recruited from the start of the trial (7 February 2014) until 4 April 2016 when protocol v4 was implemented at all study sites. Fifty-two per cent of the target number of patients had been randomised by this date.

Two different quality of life questionnaires are completed at the screening visit—the EQ-5D^33^ to assess general health outcomes and the Seattle Angina Questionnaire^34^ to assess coronary artery disease-specific quality of life. The EQ-5D is a standardised instrument for use as a measure of health outcome. Applicable to a wide range of health conditions and treatments, it provides a simple descriptive profile and a single index value for health status. The Seattle Angina Questionnaire is a disease-specific self-administered functional status measure for patients with coronary artery disease. It measures five clinically important dimensions of health in patients with coronary artery disease (anginal stability, anginal frequency, physical limitation, treatment satisfaction, quality of life) and is sensitive to clinical change over time. The (UK) English version is used.

Data are recorded on a secure dedicated electronic case report form (eCRF) and study web portal with a central database at the Robertson Centre for Biostatistics, University of Glasgow. Patients are randomised via a web portal based at the Robertson Centre for Biostatistics, University of Glasgow or interactive voice response system in a 1:1 ratio to either allopurinol or no drug to be given in addition to their usual medications. No placebo is used in the study; the comparator arm is usual care. Randomisation is stratified by primary care practice, history of MI and history of stroke, with treatment group allocated according to randomly permuted blocks of variable size. The allopurinol (oral tablet) is prescribed generically by the primary care physician.

In patients with screening visit blood results showing eGFR≥60 mL/min, allopurinol is started at 100 mg daily for 2 weeks, then titrated to 300 mg daily for 2 weeks, then 600 mg daily (given as 300 mg twice daily) if tolerated. Allopurinol is then continued at 600mg daily, given as 300mg twice daily, (or the maximum tolerated dose if lower) throughout the duration of the study for up to 5 years. In patients with screening visit blood results showing eGFR 30–59 mL/min, allopurinol is started at 100 mg daily for 2 weeks, then titrated to 300 mg daily if tolerated. Allopurinol is then continued at 300 mg daily (or the maximum tolerated dose if lower) throughout the duration of the study for up to 5 years.

Patients with screening visit blood results showing eGFR 30–59 mL/min were only included in the study from 4 April 2016 onwards following implementation of a protocol amendment that aimed to improve recruitment, enrich the study population and increase the generalisability of the results. Owing to a temporary UK shortage of allopurinol 100 mg tablet supply experienced in 2014, the protocol was amended to allow patients to start on 100–150 mg allopurinol for the first 2 weeks. The dose was then uptitrated as above. The usual starting dose of allopurinol for the majority of patients in the study was 100 mg.

Blood tests are sent to local NHS laboratories and results are checked and entered into the eCRF by study nurses. General practitioners (GPs) are alerted to any abnormal blood results. All patients are asked to report any adverse events, particularly rash or gout flares. GPs are also asked to report any serious adverse events that come to their attention during the trial. Patients experiencing a rash that could be due to allopurinol are withdrawn from allopurinol therapy. Patients withdrawing from allopurinol therapy are encouraged to still complete the planned follow-up within the study. Reasons for withdrawal from randomised therapy are recorded. Temporary withdrawals of allopurinol therapy are allowed at the discretion of a physician and reasons are documented. Dose changes are also allowed at the discretion of a physician at any time during the study (up to the maximum doses specified within the protocol). Any dose changes are recorded on the study eCRF and reasons documented. Patients undergo usual concomitant care during their participation in the trial and no interventions are prohibited during the trial.

Adverse reactions in association with allopurinol are rare in the overall treated population and mostly of a minor nature. The incidence of adverse reactions is thought to be higher in the presence of renal and/or hepatic disorders. The most common adverse reaction to allopurinol is rash (affects around 1% of patients). Usually this is a minor rash that resolves on stopping therapy. Rarely, the rash may be more serious and occasionally serious skin reactions such as Stevens Johnson syndrome or toxic epidermal necrolysis may occur. In the event of a serious skin reaction, allopurinol therapy should be stopped immediately and should not be restarted. Other possible adverse reactions to allopurinol include gastrointestinal disturbance such as nausea or vomiting, asymptomatic increases in liver function tests, hypersensitivity, angio-oedema and hepatitis. The summary of product characteristics lists other possible adverse reactions to allopurinol.

All serious adverse events are collected (whether thought to be related to randomised therapy or not). Non-serious adverse events (non-serious adverse reactions) are only collected if thought to be possibly, probably or definitely related to randomised therapy. Data on occurrence of skin rashes and gout flares are collected from all patients in the study. If any patients randomised to the non-allopurinol arm of the study later start allopurinol therapy for clinical reasons, this is recorded on the eCRF along with the reasons. At the end of the trial, patients will stop allopurinol therapy and will continue to receive usual care.

## Follow-up

### Six-week study visit

Patients randomised to allopurinol attend a study visit with a nurse 6 weeks (5–7 weeks) after starting allopurinol. At this visit, blood samples are taken for full blood count, urea and electrolytes, creatinine, eGFR and urate. Adverse events, concomitant medications, compliance with allopurinol and dose of allopurinol are recorded at this visit.

### Annual patient questionnaires

#### Adverse events, skin reactions, gout flares, compliance

Data on adverse events, skin reactions, gout flares and compliance with randomised therapy are collected by questionnaire (online, postal or telephone) at annual intervals throughout the study. Changes can also be reported to the study team at any time.

#### Quality of life

Patients complete the EQ-5D and Seattle Angina Questionnaire quality of life questionnaires after 1 year and at the end of the trial.

#### Health service usage

Data on health service usage is collected at 1 year and at the end of the trial from all participants (and from a randomly selected 25% of participants at annual intervals during the trial) by email/online where possible or otherwise by postal questionnaire or telephone. The patient is asked to report the number of visits they have made to a primary care physician, practice nurse, physiotherapist and hospital outpatient clinics over the last year. The number of hospitalisations is collected separately via the electronic record linkage system along with other outcome data.

### Record linkage

#### Record linkage for clinical events and outcomes

Record linkage for clinical events (hospitalisations, deaths and cancers) will be carried out annually for patients within the trial using national record linkage systems in Scotland (Information Services Division (ISD), NHS National Services Scotland)^35^ and England (Health and Social Care Information Centre (HSCIC)).^36^ Potential end points will be investigated further by obtaining information from medical records. End point packages will be prepared, removing identifying patient details and details of randomised treatment then adjudicated by an end point committee blinded to treatment allocation.

## Data analysis and statistical methods

### Sample size

In total, 5215 patients will be randomised to give 80% power to detect a 20% reduction in the primary CV end point for the intervention (allowing for 4% dropout for withdrawal of consent to follow-up and for non-CV deaths). A 14% event rate over 4 years average follow-up has been estimated from previous trials in similar patient groups. The study will end when 631 adjudicated primary end points have occurred.

### Primary and sensitivity analyses

Data analysis will be carried out according to a predetermined data analysis plan. The final trial data set will be available to the trial statisticians and ALL-HEART investigators. The primary analysis will be intention-to-treat. The primary outcome and its individual components (CV death, non-fatal stroke and non-fatal MI) will be analysed as cause-specific time to event outcomes using Cox proportional hazards models. Treatment effects will be estimated in the form of HRs (allopurinol vs no treatment) with 95% CIs and p values (Wald statistic). Results will be summarised graphically using cumulative incidence functions. Prespecified subgroup analyses will be carried out by investigating the effects of treatment within each subgroup with heterogeneity of treatment effect across subgroup levels assessed by fitting interaction terms to the overall Cox models. Prespecified subgroups will include patients with high or normal urate at baseline, patients with eGFR≥60 mL/min versus patients with eGFR 30–59 mL/min at screening visit, patients with CV or any hospitalisation within the year prior to entry to the study, patients aged <70 years versus those aged 70+ years. Results for other CV outcomes and all-cause mortality will be analysed in a similar manner. Time to discontinuation of allopurinol treatment will be described. Serious adverse events will be coded using MedDRA and tabulated according to system organ class and preferred term.

### Health economic assessment and analysis

The economic evaluation will assess the cost-effectiveness of adding allopurinol to usual care alone. The perspective on costs will be that of the NHS plus social services. A cost-utility analysis will be performed comparing the difference in costs (net of savings) with the difference in quality-adjusted life years estimated using a Markov modelling approach. Transition probabilities will be estimated from the trial data and used to make projections over the lifetime of the patients.

As described above, data will be collected on CV events, quality of life (using EQ-5D) and hospital admissions, as well as other resource use. We will estimate costs using sources such as Healthcare Resource Groups costs for England and Monthly Index of Medical Specialities for medicines.

Imputation techniques will be used to estimate the values for missing data based on the characteristics of the patient.

Prespecified subgroup analyses will include patients with higher than normal range uric acid at baseline versus patients with normal uric acid at baseline, patients aged <70 years versus those over 70 years, and patients with a CV or any hospitalisation in the year prior to entry to the study versus those without such a hospitalisation.

There will be uncertainty in the data, including sampling variation where data can be measured directly, and the selection of data from previous studies (eg, costs and disutility of events). A range of sensitivity analyses will be carried out including a cost-effectiveness acceptability curve, but also using threshold analysis and simpler scenario analyses as appropriate.

### Trial management

#### Trial Steering Committee

A Trial Steering Committee (TSC) oversees the conduct and progress of the trial. It includes an independent chair, independent scientific and lay members and study investigators. The chair and members of the TSC are appointed by the National Institute for Health Research (NIHR) Health Technology Assessment (HTA) programme director according to their standard procedures.

#### Independent Data Monitoring Committee

An independent Data Monitoring Committee (DMC) oversees the safety of participants in the trial. The DMC monitors unblinded comparative data and makes recommendations to the TSC on whether there are any ethical or safety reasons why the trial should not continue. The chair and members of the DMC are appointed by the NIHR HTA programme director according to their standard procedures. Planned interim data analyses will be performed for review by the DMC. The DMC will have the opportunity to make a recommendation of early stopping because of overwhelming evidence of benefit from study treatment based on interim analyses after ∼50% and 75% of the target number of adjudicated study outcomes have been observed. Overwhelming evidence of benefit is defined as evidence of benefit of allopurinol over usual care (p<0.001). Because of the conservative nature of this test, these interim analyses will have no impact on the overall sample size calculations.

#### Clinical Endpoint Committee

An independent Clinical Endpoint Committee (CEC) will adjudicate all primary end points within the trial and selected categories of secondary end points and is blinded to treatment allocation. The chair and members of the CEC are appointed by the sponsor.

#### Study sponsorship, trial monitoring, audit and quality assurance

The trial is sponsored by the University of Dundee and NHS Tayside. Email: tasctayside@nhs.net. Trial monitoring is carried out by the sponsor according to a trial-specific monitoring plan. The sponsor also undertakes audit and quality assurance activities for the trial.

### Dissemination plan

The results of the trial will be reported in peer-reviewed journals and at scientific meetings. Results will also be disseminated to guideline committees, NHS organisations and patient groups. Participants will be informed of the main trial results via a letter or newsletter and main results will be posted on the ALL-HEART study public website (http://www.allheartstudy.org). An authorship and acknowledgements policy has been approved by the TSC. No use of professional writers is intended. The protocol will be made publicly available on the NIHR website (http://www.nihr.ac.uk) and will be included as a supplementary file at the time the main trial results are published.

## Conclusions

On a background of many years of supportive evidence for the potential benefits of allopurinol in CV disease, the ALL-HEART study is a key outcome study that should answer the question of whether adding allopurinol therapy to usual care in patients with IHD aged over 60 years improves major CV outcomes. It will also provide important information on quality of life, safety of allopurinol in this patient population and health economics.
